# The Immature Stages and Natural History of *Veladyris pardalis* (Salvin, 1869) in Eastern Ecuador (Lepidoptera: Nymphalidae: Lthomiinae)

**DOI:** 10.1673/031.009.3501

**Published:** 2009-06-02

**Authors:** Harold F. Greeney, Ryan I. Hill, Wilmer Rosendo Simbaña, Grant Gentry

**Affiliations:** ^1^ Yanayacu Biological Station & Center for Creative Studies, Cosanga, Ecuador c/o 721 Foch y Amazonas, Quito, Ecuador; ^2^ 3060 Valley Life Sciences Building, Department of Integrative Biology, University of California, Berkeley, CA 94720; ^3^ Yanayacu Biological Station & Center for Creative Studies, Cosanga, Ecuador c/o 721 Foch y Amazonas, Quito, Ecuador; ^4^ Department of Ecology and Evolutionary Biology, Tulane University, New Orleans, LA 70118

**Keywords:** Andes, cloud forest, egg, larva, oviposition, pupa, Solanaceae, *Solanum*

## Abstract

We describe the immature stages and oviposition behavior of *Veladyris pardalis* (Salvin, 1869) from northeastern Ecuador. An unidentified species of *Solanum* (Solanaceae) is the larval food plant. Eggs are laid singly on leaves, stems or epiphytes growing on the host. *Veladyris pardalis* has four larval stadia, and takes 64–70 days to mature from oviposition to adult.

## Introduction

Natural history and morphological data for butterfly immature stages have proven to be useful for constructing and testing phylogenetic hypotheses (e.g., DeVries et al. 1985; Brown and Freitas 1994; Penz 1999, 2007; Freitas et al. 2002; Freitas and Brown 2004; Willmott and Freitas, 2006). A recent study of the Neotropical nymphalid subfamily Ithomiinae (Willmott and Freitas 2006) underscores the importance of acquiring data on immature stages; larval characters elucidate basal relationships that are otherwise weakly supported or obscure. Although a wealth of life history information has been published on the Ithomiinae (see Brown and Freitas 1994, Willmott and Freitas 2006 and references therein), for several key genera nothing is known, and *Veladyris* is one of those (Masters 1972, Freitas and Brown 2002, Lamas 2004).

*Veladyris* currently contains a single species, *V. pardalis*, but Lamas (2004) suggests the existence of a second as yet undescribed species. The phylogenetic position of the genus has been relatively consistent in recent molecular and morphological analyses. *Veladyris* is either considered to stand alone or, with *Velamysta* as its sister genus, to form the most basal lineage of the tribe Godyridini (Brower et al. 2006; Willmott and Freitas 2006).

*Veladyris pardalis* flies in eastern Andean cloud forest habitats, from Colombia south to Bolivia (Masters, 1972; Lamas 2004), but little else is known of its biology. Here, we describe the early stages of *V. pardalis* from eastern Ecuador, thus adding to our understanding of ithomiine biology.

## Materials and Methods

We carried out all rearing and field investigations at the Yanayacu Biological Station and Center for Creative Studies (YBS: 00°35.949 S, 77°53.403 W), located in Napo Province, in the Andes of northeastern Ecuador. The study site is located approximately five kilometers west of the town of Cosanga, and includes around 2000 hectares of primary cloud forest bordered by cattle pastures and other disturbed habitats (see Greeney et al. 2006 and Valencia 1995). We collected larvae at 2100 m, and reared them in glass jars at the onsite ambient research lab, located at 2150 m.

We observed oviposition events on 16 different occasions. We reared several larvae from the last instar to eclosion, and one individual from egg to adult. We added fresh food plant as needed, removing frass and old leaves daily. We took larval measurements on the day prior to molting.

## Results

### Oviposition behavior

Females search for oviposition sites between 10:00 and 14:00 on sunny and overcast days. They are generally inactive during periods of rain, but may continue to oviposit during light rains in the afternoon, as long as the morning itself was sunny. While searching for hostplants, females fly slowly through the understory, rarely flying above 5 m. They generally land on any broad-leafed plant below this height, pausing briefly, usually with their wings held slightly apart, to drum with their forelegs and touch their antennae to the leaf surface. Upon locating a suitable foodplant, they often fly slowly about the plant, landing on its leaves as well as those of adjacent species. Females lay 1–4 eggs (always singly), either by landing on the ventral surface of the host leaf (Figure 3) or, less frequently by landing on the leaf's edge and curling their abdomen underneath. Eggs are occasionally laid on epiphytic moss hanging from leaves of the foodplant.

### Egg (Figures 1a, 1b)
n = 7; diameter approx. 1 mm, height 1.5 mm; development time = 10 days).

White, barrel-shaped, widest around the lower third; surface covered with vertical rows of divot-like sculpturing; laid singly (n = 16); upon emergence, larvae consume entire egg shell.

### First instar (Figures 1c, 1d) n = 1; body length = 3.5–5.0 mm; development time = 6 days

Head capsule sub-quadrate, slightly broader at base, with a weak epicranial suture; head surface shiny, translucent brown, bearing a few sparse, long, pale setae; body parallel-sided, each segment slightly produced laterally; body white, with green gut contents showing through later in instar, body sparse covered with long, soft, dark setae; prothoracic shield weakly sclerotized, transparent; anal plate and A10 prolegs lightly sclerotized, dark grey; thoracic legs pale, similar in coloration to body.

### Second instar (Figures 1e, 1f)
n = 2; body length = to 7.5 mm; development time = 8 days

Head capsule more rounded than in 1^st^ instar, with a weak epicranial suture; head surface smooth, orange with dark stemmata and mandibles, a moderately dense covering of short, soft, pale setae; body round in cross-section, less produced laterally than 1^st^ instar, lateral projections only noticeable on abdomen, largest on A8; body mostly transparent, showing green dorsally from ingested material in gut; body white below spiracular line, white extending to subdorsal area on T1 and A8; A10 often appearing darker in color due to presence of internal frass pellets; body bearing minute, short pale setae, longest on A10; as larva matures, white subspiracular areas becoming less apparent and white dorsal areas on T1 and A8 becoming yellow-orange.

**Figure 1.**
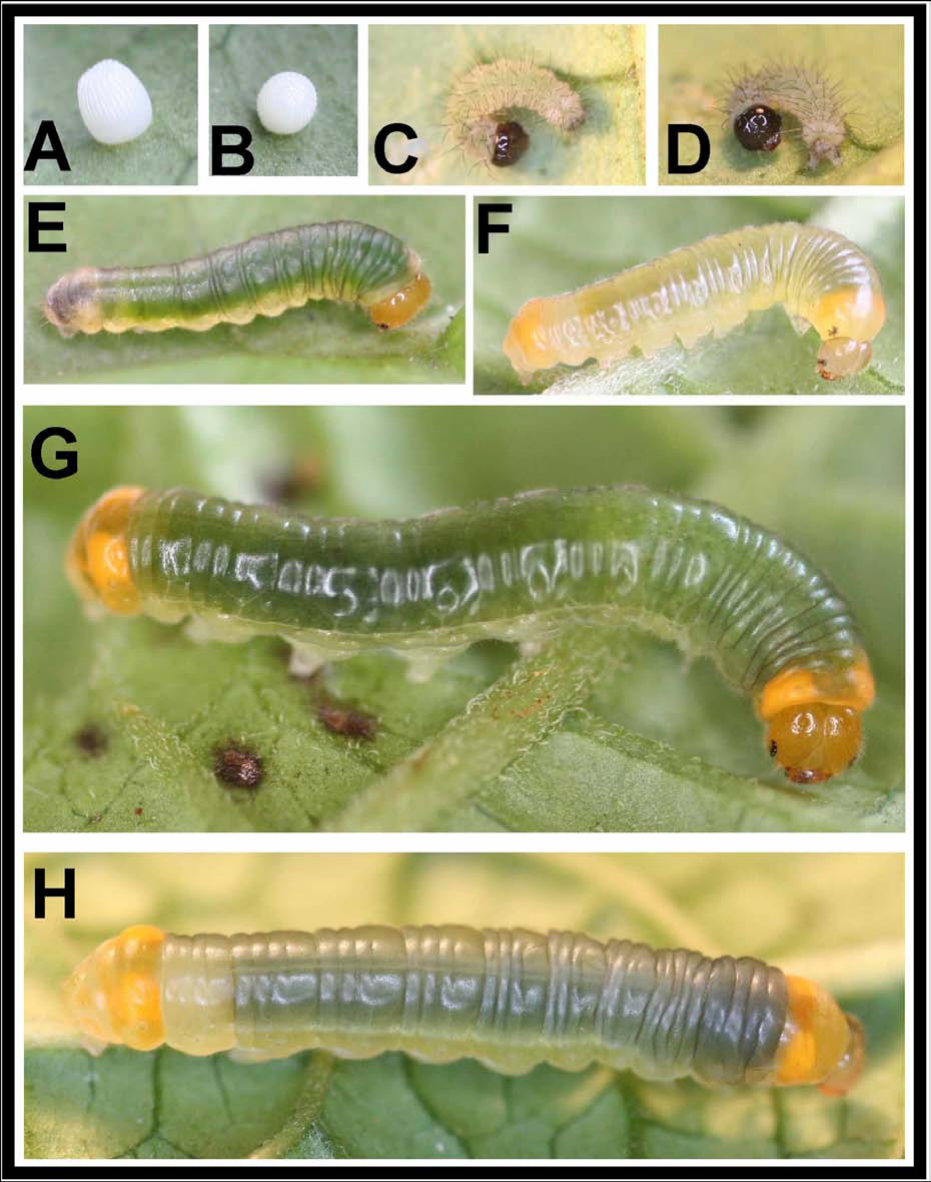
Immature stages of *Veladyris pardalis* at Yanayacu Biological Station (YBS), Napo Province, Ecuador: a-b) egg; c-d) 1^st^ instar; e) early 2^nd^ instar; f) premolt 2^nd^ instar; g) 3^rd^ instar; h) premolt 3^rd^ instar.

### Third instar (Figures 1g, 1h)
n = 5; body length = to 18 mm; development time= 10–12 days

Head and body similar to late 2^nd^ instar, T1 and A8–A9 almost entirely pale orange, T1 and A8 bearing large, distinct, darker orange subdorsal spots, lateral swellings on A8 more pronounced than in 2^nd^ instar; prolegs white, rest of body transparent, showing color of gut contents; as larva matures, a faint, longitudinal, supraspiracular white line appears on A1–A7, body develops a dull, purple-grey cast.

### Fourth instar (Figures 2a-c) n = 12; body length = to 35 mm; development time = 14–16 days including pre-pupa

**Figure 2.**
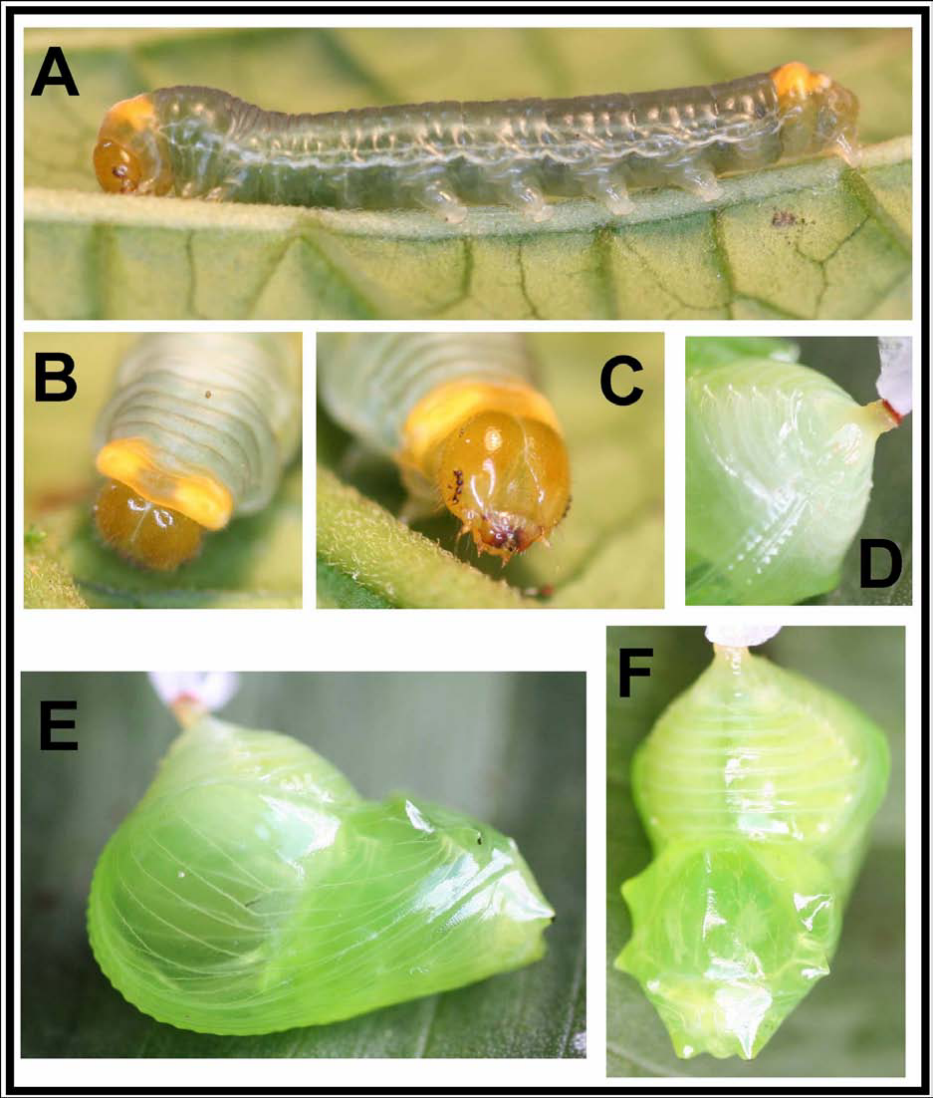
Immature stages of *Veladyris pardalis* at YBS: a) 4^th^ (final) instar; b-c) head capsule of 4^th^ instar; d) cremaster and abdominal segments of pupa; e-f) freshly formed pupa.

Head and body similar to late 3^rd^ instar; body ground color purple-grey to powdery-white (Figures 2b-c), with a thin, white subspiracular stripe from T2-A7; dorsal and lateral surfaces of T1 bright orange; a pair of large orange subdorsal spots extending across A8 and A9, A8 noticeably swollen; as larva matures, powdery-white cast disappears, body becomes transparent and shiny; orange spots on T1 become confined to dorsum; A9 portion of subterminal orange spots becoming bright white (Figure 2a).

### Pre-pupa (Figure 3)
n = 6; development time = 2–3 days

Pre-pupal larva becoming almost entirely translucent lime-green; head slightly more yellow than in 4^th^ instar; larva spins a white silk pad.

### Pupa (Figures 2d-f)
n = 2; development time = 16–18 days

Robust, pendant, angle between abdomen and thorax (“pupal angle” of Willmott and Freitas 2006) 90°; body entirely translucent lime-green, darkening slightly with age and appearing “oily”; body surface weakly reflecting a metallic gold color when viewed from various angles, never with distinct metallic patches found in other ithomiines.

## Discussion

*Veladyris* was originally placed in the Godyridini based on its genitalia (Fox, 1956). More recent systematic studies utilizing molecular and morphological data support this classification (Brower et al. 2006; Willmott and Freitas 2006). Willmott and Freitas (2006) did not identify unambiguous synapomorphies from early stages to unite the Godyridini. Our observations confirm that *Veladyns pardalis* immatures resemble other Godyridini and Dircennini. The pupa is squat and robust with a 90° pupal angle, as in *Hypoleria, Brevioleria, Dircenna* and *Hyalenna*. A weakly patterned larval body and head is typical of many Godyridini (Brown and Freitas 1994; Willmott and Freitas 2006; RIH pers. obs.). However, *Veladyris pardalis* exhibits more pronounced coloration than many godyridines, with orange extending across the dorsum on T1 and A8. The presence of a subdorsal stripe which expands dorsally onto A8 is otherwise known only in caterpillars of *Pteronymia* (Dircennini; Willmott and Freitas 2006 Character 48).

Willmott and Freitas (2006) listed several unambiguous synapomorphies uniting the Godyridini and Dircennini (excluding *Callithomia*). Like other members of this clade (node #33 of Fig. 5 in Willmott and Freitas 2006), the pupa of *V. pardalis* is a pronounced green color (their Character 63:2), the final instar has pale thoracic legs (Character 18:1), and larvae have a green venter (Character 51:2). However, *Veladyris pardalis* does not show three of the node #33 synapomorphies: 1^st^ instars have a brown head capsule (12:1); the 4^th^ instar head capsule is light brown; and larvae have lateral swellings on A8 (33:1).

Larvae of *Veladyris* show similarities, as well as differences, when compared with caterpillars of *Velamysta*, its purported sister genus (illustrated in Brown and Freitas 1994, Willmott and Freitas 2006). However, precise comparisons are somewhat ambiguous because of interspecific differences within *Velamysta: Velamysta pupilla cruxifera* appears to differ from its congener, *V. phengites phengites*, nearly as much as it does from *Veladyris pardalis*. Notable similarities between *Velamysta* and *Veladyris* include traits broadly shared across the Dircennini and Godyridini. Examples include an unmarked head capsule and body. In addition, the *Velamysta pupilla cruxifera* larva figured by Brown and Freitas (1994) has the subdorsal band on A8 expanded dorsally, somewhat similar to that of *Veladyris pardalis* and *Pteronymia* (Dircennini), but lacks the prothoracic band found in the latter two genera. First instars of *Veladyris* and *Velamysta pupilla cruxifera* have a dark head capsule (Brown and Freitas 1994), whereas that of *Velamysta phengites phengites* is pale (Willmott and Freitas 2006, figure 8K). Finally, 1^st^ instars of *Veladyris* and *Velamysta* have pale thoracic legs.

Larvae of *V. pardalis* at YBS feed on *Solanum*, thus providing support for basal placement of *Veladyris* within the Godyridini. Known Godyridini hosts are largely in the genus *Cestrum* (Solanaceae), although *Godyris* uses *Solanum* in addition to *Cestrum* (Willmott and Freitas 2006). *Solanum* is the most commonly used host among ithomiines (Willmott and Freitas 2006) and is also used by genera in the tribes Dircennini and Oleriini. Host relationships of these related genera suggest that *Solanum* is the plesiomorphic host association for the Godyridini, whereas *Cestrum-feedmg* is derived.

Interestingly, *Veladyris pardalis* is only the second species of ithomiine reported to have four larval stadia; all others have five. The other ithomiine with four instars is *Sais rosalia* from Brazil (Freitas and Brown 2002), belonging to the tribe Mechanitini (Brower et al 2006, Willmott and Freitas 2006). Instar number in *Velamysta* is currently unknown. It will be interesting to rear additional genera and species to better document the distribution of this character in the Ithomiinae.

**Figure 3.**
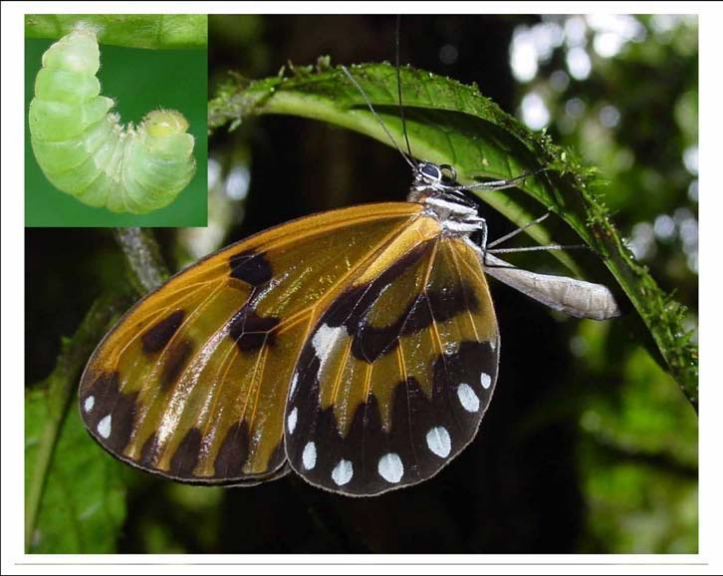
Female *Veladyris pardalis* ovipositing at YBS. Inset shows a pre-pupal larva.

### Editor's note

Paper copies of this article will be deposited in the following libraries. Senckenberg Library, Frankfurt Germany; National Museum of Natural History, Paris, France; Field Museum of Natural History, Chicago, Illinois USA; the University of Wisconsin, Madison, USA; the University of Arizona, Tucson, Arizona USA; Smithsonian Institution Libraries, Washington D.C. USA; The Linnean Society, London, England.
